# The First Case of *Cytauxzoon* spp. in Russia: The Parasite Conquers Eurasia

**DOI:** 10.3390/ani12050593

**Published:** 2022-02-27

**Authors:** Sergey V. Naidenko, Mariya N. Erofeeva, Pavel A. Sorokin, Sergey O. Gershov, Nadezhda P. Yakovenko, Alena S. Botvinovskaya, Galina S. Alekseeva

**Affiliations:** 1 Severtsov Institute of Ecology and Evolution, Leninsky pr. 33, 119071 Moscow, Russia; erofeevamariya@yandex.ru (M.N.E.); sorokin-p@yandex.ru (P.A.S.); gal.ser.alekseeva@gmail.com (G.S.A.); 2 Wildlife Nature Hospital, 101000б Chistoprudnii Bulvar, 11, 119071 Moscow, Russia; zooanest@gmail.com (S.O.G.); npya@yandex.ru (N.P.Y.); alenna70@gmail.com (A.S.B.)

**Keywords:** Amur wildcat, blood parasite, disease, felids, bobcat, piroplasm, serval

## Abstract

**Simple Summary:**

*Cytauxzoon felis* was first described more than 40 years ago in the US, later (in the 21st century) similar pathogens were detected in Eurasian and Iberian lynxes, European wildcats and domestic cats in Southern and Central Europe. Our findings have shown the previously unrecorded presence of this parasite in Russia (50 km from Moscow). We described the crucial decrease in the number of leukocytes and erythrocytes, as well as in hemoglobin concentration, throughout the captive serval’s disease, and their increase during the animal’s recovery over six months. Molecular genetic methods allowed us to detect and describe this parasite in four cat species in captivity. The analysis showed high genetic variability and high occurrence of the parasites, which suggests their presence in free-ranging domestic cats and wild felids in Russia.

**Abstract:**

Over the last two decades, *Cytauxzoon* spp. has been conquering Eurasia, although this fact has only been brought to light through recent more intensive research after the discovery of *C. manul* in Pallas’ cat. In Europe, *Cytauxzoon* was detected mainly in southern countries and later in central Europe. This pathogen has now been found in Russia for the first time (50 km from Moscow), this being the most northern sighting in Eurasia. A captive serval (*Leptailurus serval*) was found to be infected. Hematological analysis showed a crucial decrease in the number of leukocytes and erythrocytes, as well as in hemoglobin concentration. Genetic analysis confirmed the presence of *Cytauxzoon* spp. in serval blood at the beginning of the disease period. The identical pathogen was found in one bobcat at the same breeding center. Two other haplotypes of *Cytauxzoon* spp. were obtained from domestic cats at the same location, identical to the samples from Italy. One new haplotype, which was sequenced for the first time, was found in 7/7 investigated Amur wildcats (100%). The high occurrence and diversity of these pathogens suggest that they are present in free-ranging domestic cats and wild felids in Russia, and may be considered a potential threat to the endangered species. Current research shows that the genetic diversity of this pathogen may be even higher than it was suggested previously. Further genetic research is necessary to describe the diversity and phylogeny of this pathogen in Eurasia.

## 1. Introduction

The distribution of pathogens can have a significant effect on host populations, in some cases leading to epizootics [1,2,3] and the death of some individuals or a significant part of the population [4,5]. However, this is just the tip of the iceberg. Most often, pathogens, without leading directly to the death of the host organism, can provoke a decrease/change in the activity of the immune system [6] and physical condition in general [7], as well as leading to problems with reproduction [8]. Among endangered wild felines, the populations of which are relatively small (i.e., Amur tiger (*Panthera tigris altaica*) or Far-Eastern leopard (*P. pardus orientalis*)), the death or exclusion of even a few individuals from reproduction may lead to significant negative consequences for the entire population. Although the routine monitoring of the pathogens in endangered felines [9,10,11,12] is well established, the emergence of a new pathogen in a population can lead to significant changes in existing approaches.

*Cytauxzoon felis* is a protozoon of the Piroplasmida order (Theileriidae family), an intracellular blood parasite that affects primarily leukocytes, then erythrocytes. For the first time, *Cytauxzoon felis* was identified in 1976 in Missouri, USA [13], in the blood of a bobcat (*Lynx rufus*), which was supposed to be a reservoir of this pathogen. Seropositivity to *C. felis* in populations of the bobcat is estimated to be 79% [14]. Originally, it was theorized that this parasite occurs only in North America, mainly in the United States. In addition to the bobcat, it was found in the wild in the cougar (*Puma concolor*) in North America [14]. The bobcats usually remain asymptomatic and serve as reservoirs of the parasite. In contrast, infected domestic cats (*Felis catus*) usually succumb to the infection within 9 to 15 days. *Cytauxzoon felis* exists in two distinct forms: an erythrocyte phase (merozoites) and a myeloid cell phase (schizont). It is the schizogonous phase that causes the severe clinical disease in domestic cats. Infected domestic cats show clinical signs, such as pyrexia, anorexia, dehydration, depression, icterus and hepatosplenomegaly. In its normal course, the infection by this parasite leads to severe anemia, a decrease in the number of leukocytes and erythrocytes, a sharp decrease in the activity of the animal, loss of appetite, emaciation and death. Domestic cats usually die within 24 to 48 h upon presentation to veterinarians, in the presence or absence of supportive therapy [15,16,17,18,19,20]. However, recent research has indicated that this scenario can also present the other way around: bobcats may suffer and die from severe acute cytauxzoonosis [21], and in contrast, the domestic cat can recover from acute cytauxzoonosis and carry the parasite for a long period of time [22,23,24]. In this case, the domestic/stray cats may stay as the reservoir of the parasite and can be considered as a potential threat for the other species of felines in the wild and in captivity. The deaths of tigers [25,26] and lions (*Panthera leo*) [27] from acute cytauxzoonosis in captivity can serve as an example.

Cytauxzoonosis (as other piroplasmosis) is a tick-borne disease and some species of ticks play an important role in the circulation of the infection. The natural route of *C. felis* transmission to intermediate felid hosts occurs via the blood-feeding of infected ticks harboring parasite sporozoites [28,29]. For example, successful *Cytauxzoon felis* transmission studies have occurred using *Amblyomma americanum* adults, acquisition-fed as nymphs on an experimentally infected domestic cat, or *Dermacentor variabilis* adults fed as nymphs on a bobcat [30,31]. In the experimental study, *Cytauxzoon felis* was successfully transmitted to domestic cats by *A. americanum* nymphs acquisition-fed as larvae on the donor cat [32].

In Eurasia, a similar pathogen was identified in Pallas’ cat (*Otocolobus manul*) in Mongolia in 2003 [33]. Although it was named *Cytauxzoon manul* by the authors, there is still no generally accepted view of it as a separate species or as a strain of *Cytauxzoon felis*. Later this parasite was described in many European countries and this process still continues. The aim of this study is to describe the distribution of *Cytauxzoon* spp. in Eurasia with the precise analysis of the newly discovered northernmost case of cytauxzoonosis, and its similarities and differences to other cases.

## 2. Materials and Methods

### 2.1. Study Area and Objects

The description of the most northern case of cytauxzoonosis was carried out at the Severtsov Institute of Ecology and Evolution biological station in Tchernogolovka (50 km north-east from Moscow), where studies have been carried out for 25 years on the behavior and physiology of Eurasian lynxes (*Lynx lynx*) [34,35,36,37], Amur wildcats (*Prionailurus bengalensis euptilura*) [38,39,40] and domestic cats (*Felis catus*) [41,42]. In addition to these species, the Tchernogolovka station keeps bobcats, and since 2019, they have also kept caracals (*Caracal caracal*), servals (*Leptailurus serval*) and one ocelot (*Leopardus pardalis*). The husbandry conditions of these animals have been described several times before [34,38,41].

In May 2019, along with other animals, a 4.5-year-old female serval was transferred to the station. The female was obtained from a private collection and was contained for the entire summer period in the outdoor treeless enclosure of 74 m^2^, with an adjacent wire-mesh cage of 6 m^2^. The enclosure and the cage each contained a wooden house measuring 70 × 70 × 110 cm. On 6 October 2019 the female was immobilized and transferred to an indoor complex of winter enclosures. The size of the cage was 2 × 2 × 2.3 (length × width × height) m with a 70 × 100 cm shelf located 40 cm above the floor. A male serval was kept in the neighboring cage, and the winter housing also contained caracals and the ocelot. Each animal had access to the outdoor enclosure (12 m^2^), which was open when the air temperature was higher than −10 °C. Access to water was ad libitum, and the food ration included 700–900 g of chicken meat daily.

Other animals kept at Tchernogolovka were selectively tested for the presence of this parasite in the blood by molecular genetic methods, including 10 Eurasian lynxes (7 adults and 3 cubs at the age of 10 months), 23 adult domestic cats, 7 Amur wildcats and 1 bobcat. Blood was collected during the routine veterinary procedures (e.g., health check, vaccination) and analyzed as described below. From two of these animals, the samples were collected after their death (a 17-year-old bobcat and one lynx cub). In total, we tested 56% of the felids kept at the Tchernogolovka station.

### 2.2. Hematological Analysis

When transferring the serval, a blood sample was taken for hematological analysis. Later, this sample (collected on 6 October 2021) was considered as a basal level on the graphs. We collected blood using K_3_EDTA tubes. The methods of hematological analysis of the entire blood and smears process have been described earlier [40]. In the course of the disease the smears were micropsied with the Leica 5000D (Leica, Wetzlar, Germany) using 1000× magnification (100 × 10). Atypical insertions were detected and counted in blood cells and later the presence of parasites was confirmed by DNA analysis. The number of parasites per 100 red blood cells (RBC) was counted on the smears and recalculated on 1 mL of blood (based on the total number of RBC obtained from the hemoanalyzer).

### 2.3. Molecular Genetic Analysis

We stored samples (whole blood) for the genetics at −20 °C, and then performed DNA extraction with the InviMag Blood DNA Mini Kit/KF96 (Invitek Molecular, Berlin, Germany) on KingFisher Flex workstations (Thermo Fisher Scientific, Waltham, MA, USA). To detect *Cytauxzoon* spp. infection, we performed a PCR with the 5× master mix (Dialat, Moscow, Russia) and the primers 5′-TGAACGTATTAGACACACCACCT-3′ and 5′-TCCTCCCGCTTCACTCGCCG-3′ on the second internal transcribed spacer (ITS-2) of ribosomal DNA, according to Brown et al. [43]. The PCR fragments of required length were purified by excision from the 3% agarose gel using the Cleanup mini kit (Evrogen, Moscow, Russia). DNA sequencing was performed with forward and reverse primers using Big Dye 1.1 kits on an ABI 3130 genetic analyzer (Applied Biosystems, Waltham, MA, USA) in a POP7 polymer. Nucleotide sequences with a length of 232 bp were obtained from 14 animals and analyzed. Sequences are registered in NCBI (accession numbers: MZ242218–MZ242221). We compared these sequences with those of other similar *Cytauxzoon* sp. isolates available in NCBI. The percentage of identities of the ITS-2 sequences obtained in this study was compared with published sequences calculated in the Mega X program using the p-distance method. The phylogenetic trees were constructed in MEGA X by the neighbor-joining method (NJ) using the Tamura-Nei model [44].

## 3. Results

### 3.1. First Detection of Cytauxzoon spp. in Russia

**Figure 1:**
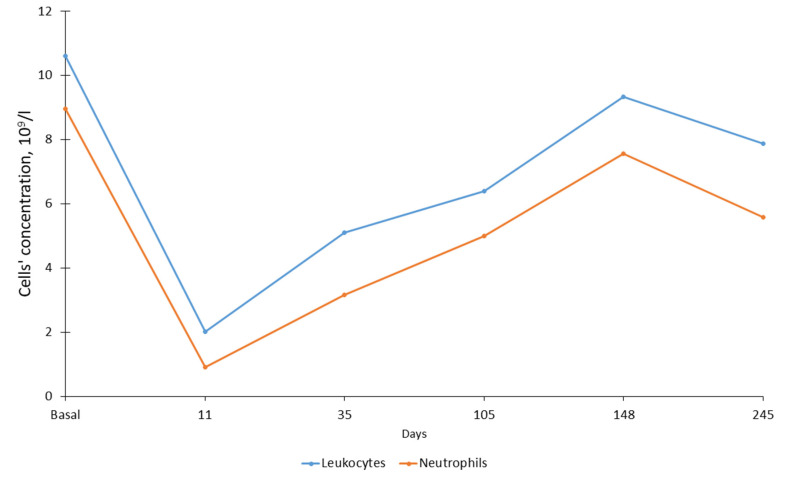
The number of white blood cells and neutrophils in serval during infection. The abscissa axe shows day after zero point.

**Figure 2:**
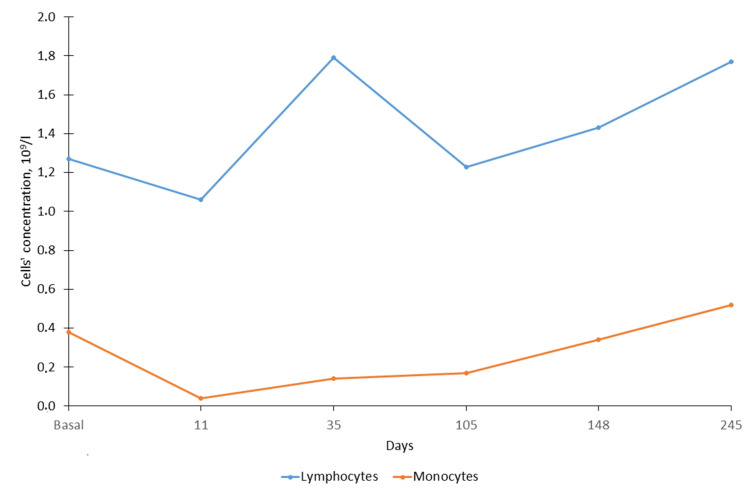
The number of monocytes and lymphocytes in serval during infection. The abscissa axe shows day after zero point.

**Figure 3:**
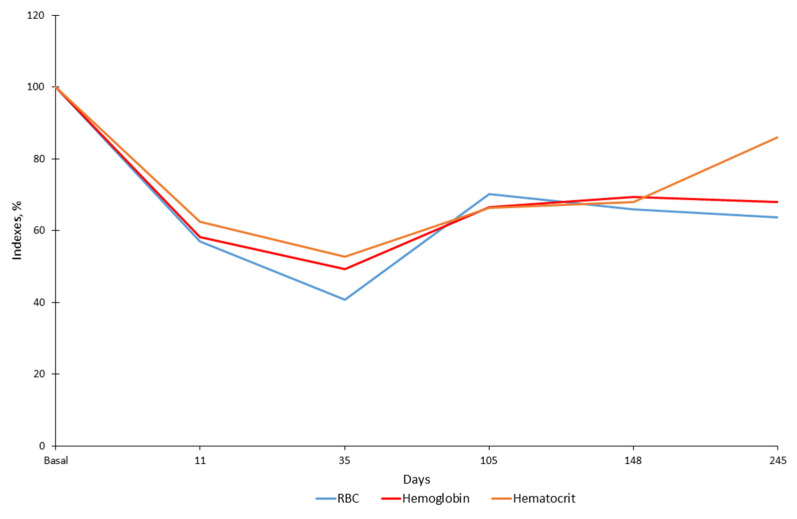
The percentage of red blood cells, hemoglobin and hematocrit in the serval during infection. The indices were calculated to basal level (6 October 2019), where these numbers were assumed to be 100%. The abscissa axe shows day after zero point.

**Figure 4:**
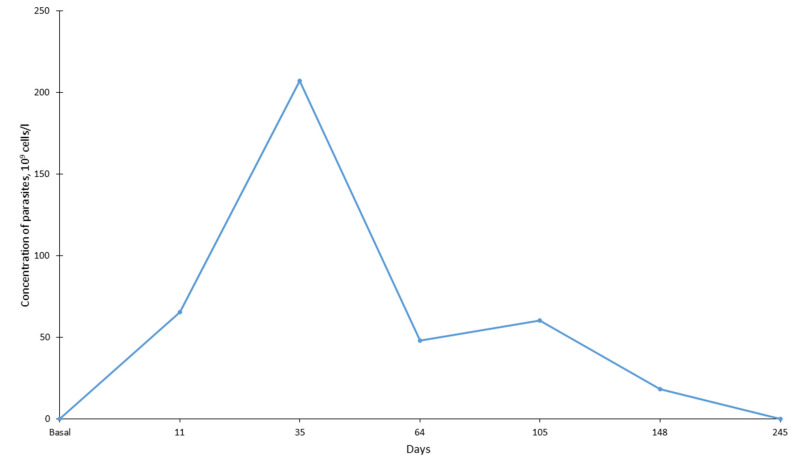
The concentration of parasites in the serval’s blood. The abscissa axe shows day after zero point.

By mid-November (16th, zero point), the serval’s behavior in captivity changed; she became inactive, moved slowly and began to regularly refuse to eat. Anesthesia of the animal and clinical examination was carried out 11 days after the zero point. Hematological analysis showed a sharp decrease in the number of white blood cells (WBC). The total number of leukocytes was almost five times less than in October at 2.2 × 10^9^ vs. 10.6 × 10^9^ cells/L (Figure 1). The total number of WBC decreased mainly because of the decrease in the number of neutrophils (almost 9 times less) (Figure 1) and monocytes (more than 9 times less) (Figure 2). The number of lymphocytes remained practically unchanged (a 20% decrease) (Figure 2). In the same period, the indicators characterizing the ability of blood to carry out oxygen transfer (the total number of red blood cells (RBC), hemoglobin content and hematocrit) almost halved (Figure 3). In the blood of the animal, the concentration of detected parasites reached 1.70/100 RBC or 65.5 × 10^9^ cells /L (Figure 4).

The first treatment was started in early December (4 December 2019), seven days after the first detection of the parasite. The animal received 70 mg of azithromycin dihydrate (Sumamed^®^, Pliva Hrvatska, Zagreb, Croatia) with food once per day for ten days, and Atovaquone 125 mg + Proguanil 50 mg (Malarone, GlaxoSmithKline, Brentford, UK) three times per day (every 8 h) for ten days. The course of treatment was completed on 13 December 2019. Blood sampling a week later (35 days after zero point) showed that the total concentration of WBC, as well as the concentration of all their forms (neutrophils, lymphocytes and monocytes), increased during the treatment period (leukocytes by 2.5 times, individual forms by 1.6–3.5 times). At the same time, the indicators for RBC, hemoglobin and hematocrit decreased by 15–28% compared to the previous sampling period. The concentration of parasites in the blood increased significantly and amounted to 7.54/100 erythrocytes or 207.3 × 10^9^ cells/L. The next course of treatment included Imidocarb dipropionate 40 mg (Pyro-Stop, Apicenna, Balashiha, Russia) and erythropoietin 500 mg (Erythropoietin, MTH, Moscow, Russia). The course of treatment was started on 30 December 2019 (44 days after the beginning of disease). Erythropoietin injections were conducted every three days (5 times). Imidocarb dipropionate was used twice with a one-week interval. One week after treatment (66th day) a blood sample was taken and showed a significant decrease (more than 5 fold) in the concentration of the parasite in the blood to 1.37/100 RBC. The serval began to eat actively.

Blood samples were collected one week after this treatment, on the 64th day. Due to the problem with the hemoanalyzer, we did not estimate the number of white and red blood cells; however, we were able to show a sharp decrease in the parasites’ number (Figure 4). The next blood sampling was performed on the 105th day. Body mass increased to 8.2 kg (13%). An increase in the number of WBC, RBC and hemoglobin by 20–50% was noted. The concentration of parasites was 1.28/100 RBC or 60.3 × 10^9^ cells/L. It turned out that the number of erythrocytes, as well as the concentration of hematocrit/hemoglobin, reached maximum levels in the animal and did not change significantly over the next five months. Although the number of WBC had increased, their concentration was significantly lower than that in autumn in a healthy animal, accounting for 60% of the total number of WBC (from 45% for monocytes to 97% for lymphocytes). However, the animal was considered rather healthy and was paired with a male two days later (on 107th day), the mating took place on 110th day.

The following samples were collected from a pregnant female on 148th day, 38 days after mating. By that point, the concentration of parasites decreased threefold in comparison to the day of the first detection. The number of RBC and hemoglobin and hematocrit levels did not change in the animal when compared to the previous sampling; however, the number of WBC increased significantly (9.34 × 10^9^ cells/L—almost reaching the level before the disease), primarily due to neutrophils (increase of 1.5 times) and monocytes (2 times). The female gave birth to a stillborn kitten 80 days after mating. The data were obtained from a video camera and the female ate the kitten several hours after birth.

The next routine examination of the animal was carried out 245 days after zero point (18 July 2020) and no parasites were detected in the animal’s blood. During this period, there was a slight decrease in the total number of WBC and neutrophils, but the number of lymphocytes and monocytes increased slightly instead. The number of red blood cells and the level of hemoglobin remained stable, while the level of hematocrit in the animal increased by about 27%.

### 3.2. An Occurrence of the Parasite in Potential Hosts and Its Genetic Differences

**Figure 5:**
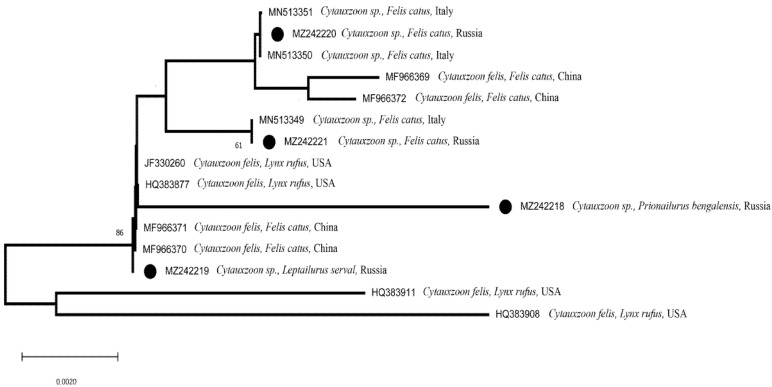
NJ tree of phylogenic relations between studied haplotypes of *Cytauxzoon* spp. and genetically similar sequences from NCBI, based on ITS-2 regions (150 bp). Haplotypes from this study are highlighted with black circles. In nodes are the results of bootstrap analysis (1000 replicas), values under 50% not shown. The scale shows the genetic distance between haplotypes.

Genetic analysis conducted with the described primers confirmed the presence of *Cytauxzoon* spp. in the blood of the serval (the obtained sequence was loaded to NCBI (accession number MZ242219)) (Table 1). The presence of the *Cytauxzoon* spp. identical to that in the serval was detected in one animal—the bobcat, which died at the age of 17 years. Thus, the presence of the pathogen of the same haplotype was confirmed in two animals (serval female and bobcat female) kept at the Tchenogolovka station. Three other haplotypes of *Cytauxzoon* spp. were obtained through sequencing of other samples. One of them, NCBI accession number MZ242218, was discovered by us for the first time (Figure 5). It was typical for Amur wildcats and was found in 100% of animals (7 of 7 tested individuals). One of them arrived from the Russian Far East two years before sampling, six others were born at the Tchernogolovka station. Two other haplotypes were obtained from domestic cats—NCBI accession number: MZ242220 from three cats and NCBI accession number: MZ242221 from two cats (respectively, 13.0% and 8.7% of the 23 tested cats). The parasite was not detected in all tested blood samples of Eurasian lynxes. All sequences analyzed in our work are unique or rare and were described only in a small number of animals with insertions of 198 bp long in the ITS-2 region, for example, NCBI accession numbers: HQ 383877 and JF 330260 from bobcats from Pennsylvania and Kentucky, USA [45]. The percentage of identities of the ITS-2 sequences obtained in this study compared with published sequences ranged from 0.00% to 94.67%. The strongest differences were found between the MZ242218 and HQ383908 sequences (Figure 5).

## 4. Discussion

### 4.1. The Current Data on Cytauxzoon spp. Distribution and Hosts

For the first time, *Cytauxzoon* spp. in Eurasia were described by US veterinarians [33]. Four Pallas’ cats were trapped in Mongolia and directly transported to Oklahoma (USA). All were examined within 10 days of their arrival. At the time of arrival, parasitemias were below 1.0%. The significance of this parasite to the health of free-ranging Pallas’ cats in Mongolia was not established. Later in Europe, this pathogen (*Cytauxzoon* spp.) was detected in domestic cats in Spain [39]. Molecular data analysis indicated that Spanish *Cytauxzoon felis* (cat isolate) were 98% identical to *Cytauxzoon* spp. from Mongolia [33] and 95% identical to African *Cytauxzoon felis*. Five years later *Cytauxzoon* spp. were detected in France in one domestic cat out of 116 tested [46,47]. The first clinical case report of *Cytauxzoon* spp. infection in a domestic cat in France was described 8 years later [48]. The animal received treatment, showed remission and recovered but one month later was brought to the vet clinic with the *Cytauxzoon* spp. infection, confirmed by PCR and smear tests. By this time the similar strain (or species) was found to be quite common in Italian domestic cats [49]. *Cytauxzoon* spp. infection was detected by 18S rRNA gene PCR in 23% and by blood smear examination in 15% of 118 tested domestic cats. The 18S rRNA gene sequences obtained were 99% identical to the *Cytauxzoon* spp. from Spanish, French and Mongolian wild and domestic cats [49], and 93% to *Cytauxzoon felis* [50].

In the last five years, the findings of this parasite in domestic cats have been reported in Portugal [51] and Switzerland [52]. In Portugal, there was an occurrence of a clinical case with a lethal outcome, despite the intensive care and treatment [51]. The parasite was genetically identical to the samples described in Spain and France [44,45]. In Switzerland this parasite was obtained from three kittens in the same litter and the transmission of the parasite with blood transfusion was confirmed for the first time [52].

Moreover, during the last 20 years *Cytauxzoon* spp. has been found in wild felines as well—in Eurasian lynx in Romania [53], in the Iberian lynxes (*Lynx pardina*) in Spain [54,55,56], and in the European forest cat (*Felis silvestris*) in Italy [57] and in Romania [53]. In the Iberian lynx, seropositivity to *Cytauxzoon* spp. was estimated at 15% [55], and the parasite was found in all four tested Eurasian lynxes [46]. In European wildcats, the serum prevalence to this parasite was 50% [53]. The distribution of the pathogen is described primarily in the eastern and southern countries of Europe, as well as in Mongolia and China [58]. Based on the data on *Cytauxzoon felis* and all other piroplasms, this parasite penetrates the cat organism (including domestic cats) only through a tick bite [24]. This suggests that infection can occur if animals are in close proximity to each other. The second option is that animals (carrier and infected) should inhabit approximately the same place, albeit at different periods of time, determined by the lifespan of the ticks that received the parasite when biting the infected animal. The distances that ticks may cross are extremely small compared to the distances of the host animals. The home range size of domestic cats is up to 2.94 km^2^ [59], of bobcats, 20.2 km^2^ [60] and of the Eurasian lynx (*Lynx lynx*), 1500 km^2^ [61]. Apparently, adult and dispersing felines are the main source of the spread of this pathogen in the wild. However, all these data and suggestions are obtained based on North American parasites. The way of transmission of European *Cytauxzoon* spp. has not been described so far. Obtaining these data is a crucial goal for the understanding of the life cycle of these parasites in Europe.

In Russia, this pathogen has never been reported before, neither in the south of the European part (Caucasus, inhabited by the Central Asian leopard (*Panthera pardus saxicolor*) as well as Eurasian lynx, European wildcat, domestic cat), nor in the border areas with Mongolia (snow leopard (*Panthera uncia*), Pallas’ cat, domestic cat).

### 4.2. The First Detected Case in Russia

We assume that the infection of the female serval occurred at the Tchernogolovka station. The first blood sampling, carried out in October 2019, did not reveal the presence of the parasite in this female (neither by microscopy of the blood smears nor by PCR). However, already in November we noted a sharp deterioration in blood parameters. It is difficult to assess what was the source of the pathogen. The design of the open-air cage complex practically excludes the contact of animals with stray cats. At the same time, the North American species (*Cytauxzoon felis*) is carried mainly by ticks, which can travel distances of tens of meters. The bobcat, a confirmed host of the identical parasite, was kept 70 m from the serval enclosure, which was not an insurmountable obstacle for the tick. In addition, the transfer of ticks on the keepers’ clothes cannot be ruled out. However, in this case the disease manifested itself approximately a month after the bite, which is not typical for most cases of the *Cytauxzoon* spp. infection, which usually develops much faster [15,20]. An alternative is the possibility of infection with the pathogen from animals transferred with the serval to the winter enclosure (an ocelot, a serval male and six caracals). Although genetic analysis of the blood of these animals for the presence of the pathogen was not carried out, the analysis of blood smears and the observation of the physical condition of the animals did not reveal signs of infection. Thus, there are two unproven hypotheses of serval infection—either a long (about a month) incubation period of the disease, or the latent course of the infection in a number of felines. From another point of view, as we know, the transmission routes, including the vector tick, as well as the clinical signs, are not well resolved for the European *Cytauxzoon* spp. Thus far, it is difficult to suggest another way of pathogen transmission because all infections by piroplasms are related to ticks. However, further studies are necessary in order for us to understand the transmission routes for the European species of *Cytauxzoon*.

This is the first known case of the discovery of *Cytauxzoon* spp. in a serval, which does not allow an effective assessment of the typical course of the disease. Its course and mortality differ significantly in domestic cats and lynxes/bobcats. The latter, apparently, may be asymptomatic, the animals acting as reservoirs of the pathogen [14], but see [24]. Despite the described isolated cases of disease and death in other wild feline species [14,25,26], it is not possible to assess the danger of the pathogen for these species and the typical course of the disease. The two forms/phases were described for *Cytauxzoon felis*: the erythrocyte phase (merozoites) in blood and the myeloid cell (schizogonus) phase. The latter causes the most severe consequences for its hosts. Thus far, the schizogenous phase has not yet been identified in European *Cytauxzoon* spp. The serval is a species that is evolutionarily closer to the lynx than to the domestic cat [62], which theoretically may affect the course of the disease. However, in this clinical case, the animal suffered from this disease severely. At the same time, despite the two types of treatment, parasites in the serval’s blood were recorded for at least five months. Nothing is known about the effect of this pathogen on pregnancy, however, the female serval gave birth to at least one stillborn kitten. By the middle of pregnancy, the concentration of WBC in the serval reached normal, which can partly be explained by the stimulation of the female’s immunity by embryos during this period [42].

In addition, this is the first confirmed case of detection of this pathogen in Russia. Based on the confirmed distribution of this pathogen outside Russia, it can be expected to occur in domestic and wild cats in the Caucasus region and in Transbaikalia (along the border with Mongolia), however, the parasite was identified in the Moscow region. One study [63] mentions that *Cytauxzoon* was discovered by PCR in one Amur wildcat and in one Asiatic badger (*Meles leucurus*) in the Russian Far East, but it did not describe the year, precise region and methods of the study.

### 4.3. The Diversity of Cytauxzoon spp. in the Animals at the Biological Station Tchernogolovka

The Tchernogolovka biological station has, for many years, supported a large colony of Eurasian lynxes that can act as carriers and reservoirs of the pathogen, however, the presence of the pathogen was not detected in any of these animals. The only animal of those tested that was positive for the pathogen, was the old bobcat obtained in 2004 from Moscow Zoo. Surprisingly, the haplotype of the parasite (NCBI accession number: MZ242219) was similar to that which was described for the domestic cats in China [58].

We also found three more haplotypes (or possibly species) of *Cytauxzoon* spp. in cats at the Tchernogolovka station. Two sequences of *Cytauxzoon* spp. haplotypes, obtained from domestic cats (NCBI accession numbers: MZ242220 and MZ242221), were identical to ITS-2 sequences available in NCBI (accession numbers: MN513351, MN513350 and MN513349), which were obtained in Italy. Although the authors did not present the link with the publication, we assume that they were obtained from domestic cats [54,55] and European wildcats [62]. Both of these haplotypes were found at the Tchernogolovka station in domestic cats. Another new haplotype was found in Amur wildcats. The Amur wildcats were kept far away from the domestic cats (about 200 m) for several years, which likely prevented the transfer of parasites between these two colonies. Alternatively, there is supposed species-specificity of host–parasite relations in *Cytauxzoon*. The percentage of infected animals was higher in the Amur wildcat than in the other two species (domestic cat and Eurasian lynx).

During the last two decades, *Cytauxzoon* spp. has been found in Eurasia and a number of studies have described its presence in many European countries, Mongolia and China [28,51,52,53,54,55,56,57,58]. It was shown that this parasite is different from the North American *Cytauxzoon felis* and more similar to the *Cytauxzoon manul*. These findings belong mainly to the southern latitudes (Mongolia, China, Southern Europe). Only in the last few years has this parasite been found in central Europe (Switzerland and Germany [52,64]). However, the recording of *Cytauxzoon* spp. in the surroundings of Moscow is the most northern finding of the pathogen in Eurasia. This record was obtained in captivity, however, the infection of the serval suggests tick transfer (like in other piroplasms) of the pathogen at this latitude as well. Moreover, the wide distribution of ticks in northern areas of Russia and the wide distribution of Eurasian lynxes and stray cats, suggest that *Cytauxzoon* spp. should occur at higher latitudes as well. It is probable that the lack of information here can be explained by the absence of studies in these areas.

The high diversity of this pathogen was detected at the Tchernogolovka station, as well as its occurrence in different cat species (for example, one haplotype was obtained only from an Amur wildcat, but 100% of animals had this parasite). This partial exclusiveness (host–species specificity, but the same haplotype was detected in bobcats and servals) led us to the theory regarding the high diversity of these haplotypes/species and the difficulties in interspecies pathogen transfer. Gallusova et al. [53] suggested this idea several years ago. A more detailed study of the health status of wild and stray cats over large areas in Russia will definitely allow us to describe the genetic diversity of this pathogen. A recent study [64] suggested that *Cytauxzoon* in Europe includes at least three different species. One of them (*C. europaeus*) was described both in the European wildcat and Eurasian lynxes, but two others were collected only from the European wildcats (*C. otrantorum* and *C. banethi*). One of them was found only in Romania [64]. Although, we used other genetic primers in our project, which makes our data incompatible with this study. However, the high occurrence and high haplotype diversity of *Cytauxzoon* spp. at the Tchernogolovka station suggest that this pathogen may occur in wild and domestic felids in Russia. Further studies of the pathogen and its genetic diversity are necessary to assess its distribution in Russia and the degree of threat to cats, both in various breeding centers and in the wild, primarily in the southern regions of the country, in order to identify the species of these pathogens and their pathogenicity to domestic cats and wild felids.

The latest findings of the *Cytauxzoon* spp. in Europe may give an impression that the parasite has dispersed intensively in the European region in the last two decades. Following the order of publication, the pathogen seems to have spread to north-eastern Europe (from Spain [54] and Italy [49] to Germany [52] and Romania [64]), and has now been discovered in Russia, near Moscow. The parasite appears to be conquering the continent. However, increasing the intensity of the studies of this pathogen after the first finding in Europe may lend this explanation more credence. The last findings of the pathogen in the samples of European wildcats collected at the end of the 20th century confirm the presence of this parasite in France during that period [65]. *Cytauxzoon* spp. are widely distributed in Europe, but our knowledge about it is relatively scarce in contrast to *C. felis*. The phylogeny of European *Cytauxzoon* spp. [64], its distribution, the vectors of the pathogen transmission and the details of its life cycle (the existence of the schizont stage) create a necessity for further investigation of this parasite over the whole range.

## 5. Conclusions

The latest studies indicated the presence of *Cytauxzoon* spp. in Eastern and Southern Europe. This study described the first case of infection by this parasite at the biological station in Russia in a captive serval (*Leptailurus serval*), the most northern case of pathogen detection. It also showed a clinical picture of the animal recovering from the disease. Hematological analysis showed a crucial decrease in the number of leukocytes and erythrocytes, as well as in hemoglobin concentration, which recovered only six months later. The peak of parasite concentration was observed two and a half months after infection. Genetic analysis confirmed the presence of the identical strain of *Cytauxzoon* spp. in one bobcat at the same breeding center. Three more strains of *Cytauxzoon* spp. were obtained there from domestic cats and Amur wildcats. Further genetic analysis is necessary to validate these strains with the new classification of *Cytauxzoon* spp. proposed by Panait with co-authors [64]. The high occurrence and diversity of these pathogens in captivity suggest that they are present in free-ranging domestic cats and wild felids in Russia, and may be considered a potential threat to the endangered felid species.

## Figures and Tables

**Table 1:** Tested felids at the biological station Tchernogolovka for *Cytauxzoon* spp. (ad—adults, sad—subadults). The strains are named according to the text above.

№	Species	Name	Age	Test	Strain
1	Amur wildcat	Aralia	ad	positive	MZ242218
2	Amur wildcat	Ayuna	ad	positive	MZ242218
3	Amur wildcat	Dinya	ad	positive	MZ242218
4	Amur wildcat	Malta	ad	positive	MZ242218
5	Amur wildcat	Munko	ad	positive	MZ242218
6	Amur wildcat	Sguschenka	ad	positive	MZ242218
7	Amur wildcat	Ugra	ad	positive	MZ242218
8	Bobcat	Alamista	ad	positive	MZ242219
9	Domestic cat	Astrid	ad	negative	
10	Domestic cat	Bjorn	ad	positive	MZ242220
11	Domestic cat	Celsium	ad	negative	
12	Domestic cat	Dimka	ad	negative	
13	Domestic cat	Elka	ad	negative	
14	Domestic cat	Finik	ad	positive	MZ242220
15	Domestic cat	Fishka	ad	negative	
16	Domestic cat	Izum	ad	negative	
17	Domestic cat	Jivs	ad	negative	
18	Domestic cat	Kleo	ad	negative	
19	Domestic cat	KMeridi	sad	negative	
20	Domestic cat	Konor	ad	negative	
21	Domestic cat	LN	ad	negative	
22	Domestic cat	Merida	ad	negative	
23	Domestic cat	Milka	ad	negative	
24	Domestic cat	Paslkal	ad	negative	
25	Domestic cat	Pishka	ad	positive	MZ242220
26	Domestic cat	Rioha	ad	positive	MZ242221
27	Domestic cat	Riska	ad	positive	MZ242221
28	Domestic cat	Snezhka	ad	negative	
29	Domestic cat	Sunny	ad	negative	
30	Domestic cat	Torvi	ad	negative	
31	Domestic cat	Zlata	ad	negative	
32	Lynx	Botsman	ad	negative	
33	Lynx	Freya	ad	negative	
34	Lynx	Koritsa	ad	negative	
35	Lynx	Ovsyanka	ad	negative	
36	Lynx	Pirat	ad	negative	
37	Lynx	Suchok	ad	negative	
38	Lynx	Vetka	ad	negative	
39	Lynx	KNobli1	sad	negative	
40	Lynx	KNobli2	sad	negative	
41	Lynx	KNobli3	sad	negative	
42	Serval	Vega	ad	positive	MZ242219

## Data Availability

May be received from the corresponding author (snaidenko@mail.ru) upon request.
